# Supplementary material for: Prevalence and Predictors of Using Antibiotics without a Prescription in a Pediatric Population in the United States
Source: Antibiotics (Basel). 2023 Mar 1;12(3):491. doi: 10.3390/antibiotics12030491 (PMC10044616; doi:10.3390/antibiotics12030491)
Supplement: Supplementary file 1 [file antibiotics-12-00491-s001.zip › Supplemental File S1.pdf]

QUESTIONNAIRE

Throughout this survey, I will often use the word antibiotic(s). An antibiotic is a medication that is used to fight infections. Please feel free to ask me to repeat or clarify what an antibiotic is at any point during this survey.
Note: A Research Coordinator will show the list of most commonly used pediatric antibiotics in the United States and Latin American countries (brand names and generic names) with photos as a reference.

__ __ __

1. Has any child in your household taken antibiotics in the last 12 months (including current use)?

☐ Yes ☐ No ☐ Don't remember

1A. If **YES**, tell me the name of the antibiotic(s), the reason (symptoms, illness) for taking them, and for how long they were taken. .
If the caregiver does not remember, please write in any applicable column, *do not remember*.

Name of antibiotic (see the list)	What were the antibiotics used for?	Number of days used	Prescribed by a doctor, nurse, dentist, or hospital	Without prescription, directly from a market or store in the U.S.	Without prescription, from another country	Antibiotics you had leftover from previous prescription(s)	Without prescription, from the Internet	From antibiotics prescribed for child's sibling or another child	Other source, please explain
1.			☐	☐	☐	☐	☐	☐	
2.			☐	☐	☐	☐	☐	☐	
3.			☐	☐	☐	☐	☐	☐	
4.			☐	☐	☐	☐	☐	☐	
5.			☐	☐	☐	☐	☐	☐	
6.			☐	☐	☐	☐	☐	☐	
7.			☐	☐	☐	☐	☐	☐	

2. Excluding antibiotics your child is currently using, do you have antibiotics at home right now?

☐ Yes ☐ No ☐ Don't remember

2A. If **YES**, can you tell me the name of the antibiotic(s) you have and tell me how you got each antibiotic. A list of the most commonly used antibiotics is given as a reference. Please review the list.

Name of antibiotic (see the list)	Prescribed by a doctor, nurse, dentist, or hospital	Without prescription, directly from a market or store in the U.S.	Without prescription, from another country	Without prescription, from the Internet	From antibiotics prescribed for child's sibling or another child	Other source, please explain
1.	☐	☐	☐	☐	☐	
2.	☐	☐	☐	☐	☐	
3.	☐	☐	☐	☐	☐	
4.	☐	☐	☐	☐	☐	

3. In general, would you give antibiotics to your child without contacting a doctor/nurse/dentist/hospital?

☐ Yes ☐ Maybe ☐ No ☐ Don't know

3A. If **YES** or **MAYBE**, tell me any symptoms or illnesses for which you would use antibiotics for your child without contacting a doctor or a hospital (*the coordinator will mark **ALL** boxes that apply*).

Runny nose/cold	Cough	Bronchitis	Sore throat	Sinus infection	Fever	Flu	Ear Infections/ear pain	Toothache	Diarrhea	Urinary tract infections/cystitis	Other illnesses
☐	☐	☐	☐	☐	☐	☐	☐	☐	☐	☐	☐

Please describe any other infections/illnesses that were not listed above.

……………………………………………………………………………………

4. What is your sex?
- ☐ Male
- ☐ Female
- ☐ Other:_________

5. What is your age (in years)?

____ Years

6. Do you consider yourself to be Hispanic/Latino?
- ☐ Yes
- ☐ No

7. Which category best described your race?
- ☐ Black or African American
- ☐ White
- ☐ Declined
- ☐ Other, please specify: _____________

8. What is the highest level of education you completed?
- ☐ Never attended school
- ☐ Grades 1 through 5 (Elementary)
- ☐ Grades 6 through 8 (Middle School)
- ☐ Grades 9 through 11 (Some High School)
- ☐ Grades 12 or GED (High School graduate)
- ☐ College 1 year to 3 years (Some college or technical school)
- ☐ College 4 years or more (College graduate)

9. How many children under the age of 18 live in your household?

____

10. What was the total annual income in your household in the past year?
- ☐ Less than $20,000
- ☐ $20,000 or more but less than $40,000
- ☐ $40,000 or more but less than $60,000
- ☐ $60,000 or more but less than $100,000
- ☐ More than $100,000
- ☐ Don’t know/prefer not to say

11. How often do you have problems learning about your medical condition because of difficulty understanding written information?
- ☐ Never ——— Go to Question #13
- ☐ Occasionally
- ☐ Sometimes
- ☐ Often
- ☐ Always

12. Was this because the material was not written in your native language?
- ☐ Yes
- ☐ No

13. How confident are you filling out medical forms by yourself?
- ☐ Extremely
- ☐ Quite a bit
- ☐ Somewhat
- ☐ A little bit
- ☐ Not at all

14. How often do you have someone help you read clinic or hospital materials?

- ☐ Never ⟶ Skip Question #15
- ☐ Occasionally
- ☐ Sometimes
- ☐ Often
- ☐ Always

15. Was this because the material was not written in your native language?

- ☐ Yes
- ☐ No

You have completed the questionnaire. Thank you for your participation!
