# Supplementary material for: Prevalence and Predictors of Using Antibiotics without a Prescription in a Pediatric Population in the United States
Source: Antibiotics (Basel). 2023 Mar 1;12(3):491. doi: 10.3390/antibiotics12030491 (PMC10044616; doi:10.3390/antibiotics12030491)
Supplement: Supplementary file 1 [file antibiotics-12-00491-s001.zip › Supplemental File S2.pdf]

List of Most Commonly Used Antibiotics for Children

Note: This table is intended to be used as a guide to help identify antibiotics that have been previously taken. It lists examples of the most commonly used brand name and generic antibiotics. The antibiotic images are not scaled to their actual size and can vary in shape, flavor, and/or color compared to those presented.

**Amoxicillin**		**Amoxicillin-Clavulanate**		**Sulfamethoxazole/ Trimethoprim**	
Amoxil	Amoxicillin	Augmentin	Amoxicillin-Clavulanate	Bactrim	Sulfamethoxazole / Trimethoprim
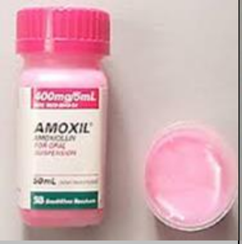	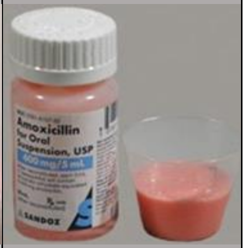	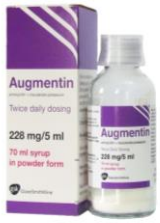	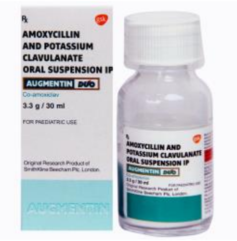	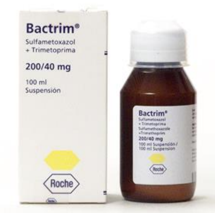	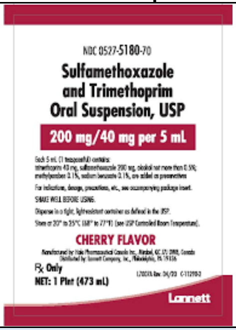
**Clindamycin**		**Cefdinir**		**Cefixime**	
Cleocin	Clindamycin	Omnicef	Cefdinir	Suprax	Cefixime
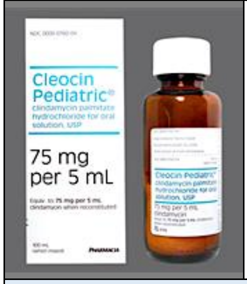	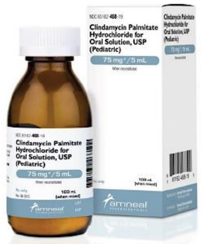	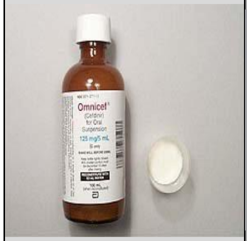	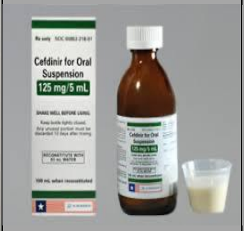	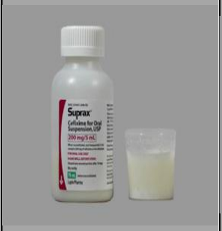	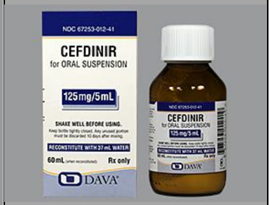
**Cephalexin**		**Antibiotic Flavoring and Coloring**			
Keflex	Cephalexin	Common Flavors			
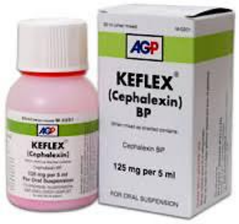	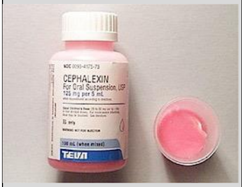	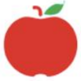 Apple 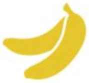 Banana	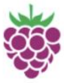 Grape 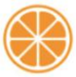 Orange	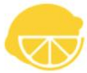 Lemon 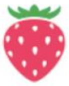 Strawberry	

LISTA DE LOS ANTIBIÓTICOS MAS COMUNMENTE USADOS PARA NIÑOS

Nota: Esta tabla está destinada a ser utilizada como una guía para ayudar a identificar los antibióticos que se han tomado anteriormente. Enumera ejemplos de antibióticos de marca y genéricos más utilizados. Las imágenes de antibióticos no se escalan al tamaño correcto y pueden variar en forma, sabor y/o color en comparación con las presentadas.

**Amoxicilina**		**Amoxicilina/ Ácido Clavulánico**		**Sulfamethoxazole/ Trimethoprima**	
Amoxil	Amoxicilina	Augmentin	Amoxicilina / Ácido Clavulánico	Bactrim	Sulfamethoxazole / Trimethoprima
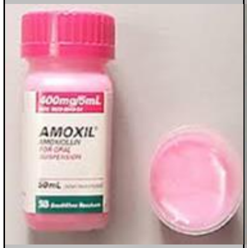	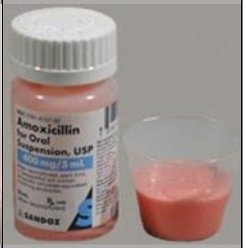	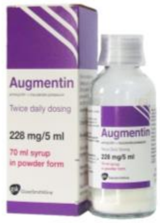	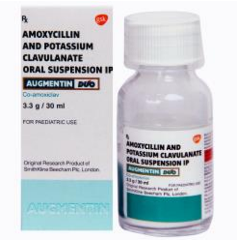	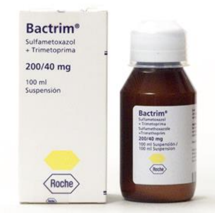	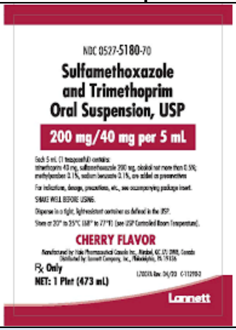
**Clindamicina**		**Cefdinir**		**Cefixima**	
Cleocin	Clindamicina	Omnicef	Cefdinir	Suprax	Cefixima
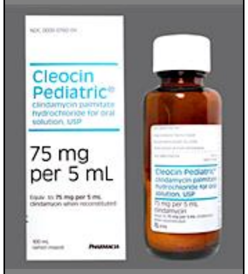	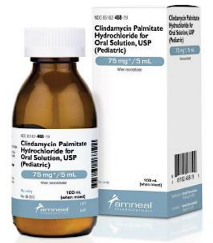	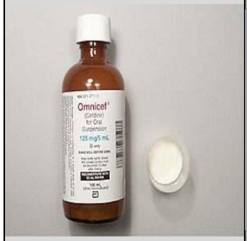	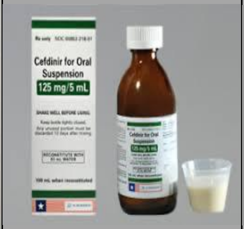	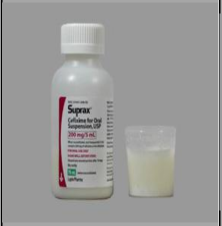	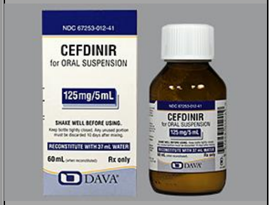
**Cefalexina**		**Aromatizantes y Colorantes de Antibióticos**			
Keflex	Cefalexina	Sabores Comunes			
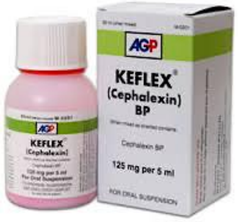	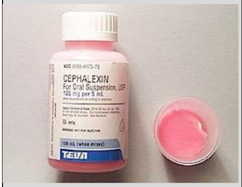	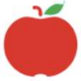 Manzana 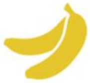 Plátano	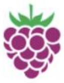 Uva 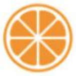 Naranja	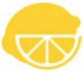 Limón	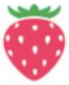 Fresa
